# Personal

**Published:** 1889-12

**Authors:** 


### Personal.

Dr. Jacob Goldberg announces that he will hereafter limit his practice to diseases of the eye and ear. He has spent some time with Drs. Knapp and Moore, in New York, preparing himself for his chosen specialty, and may be consulted at 145 Cedar street.

Surgeon William S. Tremaine, U. S. A., has been relieved from temporary duty at Fort Leavenworth, Kan., and ordered to return to his home in this city.

Assistant Surgeon Louis M. Maus, U. S. A., has been relieved from duty at Fort Porter, in this city, and ordered to Fort Stanton, New Mexico.

Assistant Surgeon Edwin F. Gardner, U. S. A., is relieved from duty at Fort Lewis, Col., and ordered to duty at Fort Porter, Buffalo, N. Y.

Dr. Bache McE. Emmet and Dr. Horace T. Hanks have been appointed Surgeons to the Woman's Hospital of the State of New York, the first to succeed Dr. James B. Hunter, deceased, and the second in place of Dr. C. C. Lee, resigned.

